# Clinical validation of the use of prototype software for automatic cartilage segmentation to quantify knee cartilage in volunteers

**DOI:** 10.1186/s12891-021-04973-4

**Published:** 2022-01-03

**Authors:** Ping Zhang, Ran Xu Zhang, Xiao Shuai Chen, Xiao Yue Zhou, Esther Raithel, Jian Ling Cui, Jian Zhao

**Affiliations:** 1 Department of Radiology, The Third Hospital of Hebei Medical University, Hebei Province Biomechanical Key Laboratory of Orthopedics, Shijiazhuang, Hebei China; 2 MR Collaboration, Siemens Healthineers Ltd., Shanghai, China; 3 Siemens Healthcare, Erlangen, Germany

**Keywords:** MRI, Cartilage segmentation, Automatic segmentation, Manually corrected

## Abstract

**Background:**

The cartilage segmentation algorithms make it possible to accurately evaluate the morphology and degeneration of cartilage. There are some factors (location of cartilage subregions, hydrarthrosis and cartilage degeneration) that may influence the accuracy of segmentation. It is valuable to evaluate and compare the accuracy and clinical value of volume and mean T2* values generated directly from automatic knee cartilage segmentation with those from manually corrected results using prototype software.

**Method:**

Thirty-two volunteers were recruited, all of whom underwent right knee magnetic resonance imaging examinations. Morphological images were obtained using a three-dimensional (3D) high-resolution Double-Echo in Steady-State (DESS) sequence, and biochemical images were obtained using a two-dimensional T2* mapping sequence. Cartilage score criteria ranged from 0 to 2 and were obtained using the Whole-Organ Magnetic Resonance Imaging Score (WORMS). The femoral, patellar, and tibial cartilages were automatically segmented and divided into subregions using the post-processing prototype software. Afterwards, all the subregions were carefully checked and manual corrections were done where needed. The dice coefficient correlations for each subregion by the automatic segmentation were calculated.

**Results:**

Cartilage volume after applying the manual correction was significantly lower than automatic segmentation (*P* < 0.05). The percentages of the cartilage volume change for each subregion after manual correction were all smaller than 5%. In all the subregions, the mean T2* relaxation time within manual corrected subregions was significantly lower than in regions after automatic segmentation (*P* < 0.05). The average time for the automatic segmentation of the whole knee was around 6 min, while the average time for manual correction of the whole knee was around 27 min.

**Conclusions:**

Automatic segmentation of cartilage volume has a high dice coefficient correlation and it can provide accurate quantitative information about cartilage efficiently without individual bias.

Advances in knowledge: Magnetic resonance imaging is the most promising method to detect structural changes in cartilage tissue. Unfortunately, due to the structure and morphology of the cartilages obtaining accurate segmentations can be problematic. There are some factors (location of cartilage subregions, hydrarthrosis and cartilage degeneration) that may influence segmentation accuracy. We therefore assessed the factors that influence segmentations error.

## Backgroud

Biochemical cartilage information plays an even more important role than morphology in detecting early cartilage change. Developments in magnetic resonance imaging (MRI), such as three-dimensional (3D) quantitative MRI, allow for sensitive analysis of cartilage morphology. Quantitative parameters derived by MRI, such as T2* relaxation time, T2 relaxation time and T1rho can reflect biochemical changes in articular cartilage and can detect initial stages of cartilage degeneration [1–4]. According to some recent reports, T2* relaxation demonstrates a similar response in the assessment of articular cartilage and cartilage repair tissue [5–7]. Three-dimensional double-echo steady-state (3D-DESS) sequence is a common MRI sequence for morphological imaging of musculoskeletal diseases. Its reported sensitivity, specificity and accuracy for detection of cartilage lesions are 96.7, 75, and 93.7%, respectively [8]. It is usually used for the diagnosis of cartilage lesions [9]. Combining T2* mapping and 3D morphological imaging, therefore, has potential to enhance the assessment of articular cartilage.

Cartilage quantitative parameters, including cartilage volume and thickness, can be obtained noninvasively and can be derived automatically once a segmentation of a high-resolution 3D MRI sequences is available. These tools are valuable in a clinical setting because they can be used to quantitatively assess cartilage and save time in post-processing. Unfortunately, automated segmentation software can result in errors in segmentation of cartilage in some subregions [10]. Of the various cartilage segmentation algorithms, an approach proposed by J. Fripp et al. has been shown to be comparable or superior to other published automatic algorithms [11]. The deep learning algorithms would probably have high accuracy in cartilage segmentation, but it did not exceed individual network performance in cartilage thickness accuracy and voting ensembles [12]. However, there is no report on the relationship between the accuracy of deep learning method in cartilage segmentation and articular effusion and cartilage degeneration. This approach relies on a segmentation hierarchy, using machine learning to train three-dimensional active shape models to segment bone. Cartilage is segmented afterwards, by using a deformable model including the expected cartilage thickness and patient-specific tissue estimation. Recently, a study demonstrated that an approach that combines a deep convolutional neural network (CNN) and 3D simplex deformable modeling is useful for performing rapid and accurate cartilage and bone segmentation within the knee joint [13]. However, that segmentation algorithm relies on accurate recognition of the boundary between tissues, which is easily influenced by hydrarthrosis and by edge blur caused by cartilage degeneration. The segmentation accuracy of that method remains to be clinically validated.

There are some factors (location of cartilage subregions, hydrarthrosis and cartilage degeneration) that may influence the accuracy of segmentation. We therefore assessed the influence factors for the error of segmentations using the approach from Fripp et al. (2010), based on 3D DESS and T2* relaxation time data. Cartilage quantitative parameters, including cartilage volume and thickness, can be obtained noninvasively and derived automatically once a segmentation of a high resolution 3D MRI sequence is available. Accuracy of automatic cartilage segmentation was assessed by comparing results to those from manually corrected contours of knee cartilage. Moreover, mean T2* relaxation time of these cartilage subregions were also measured.

## Methods

We examined 32 right knees of 32 volunteers, each of whom underwent MRI examinations. The volunteers included 13 males and 19 females, aged 21 to 37 years (mean 27.5 ± 5.2 years). Their body mass index (BMI) was between 17 and 28 kg/m^2^ (mean 21.9 ± 2.5 kg/m^2^). This study was approved by the ethics committee of our hospital (2019–003-1), and all participants provided written informed consent. Inclusion criteria were: (1) age 18–40 years; (2) BMI < 28 kg/m^2^; (3) without knee infection, trauma, or surgery; and (4) without chronic diseases. Exclusion criteria were (1) knee injury; (2) morphological damage to articular cartilage; (3) knee pain or other positive symptoms; and (4) contraindication for MRI examination.

Scans were performed on a 3 T MR scanner (MAGNETOM Verio, Siemens Healthcare, Erlangen, Germany) using the 8-channel knee coil. The 3D knee images were obtained to show the high-resolution morphology using a 3D-DESS sequence with selective water excitation. The imaging parameters were: voxel size 0.63 × 0.63 × 0.68 mm^3^, TE 5.17 ms, TR 14.45 ms, flip angle 25°, matrix: 256 × 256 × 240, FOV: 160 × 160 mm^2^. The sagittal T2* maps were obtained utilizing 5 echoes for the fit: TE = 4.36, 11.9, 19.44, 26.98, 34.52 ms, TR = 1340 ms, FOV = 160.0 × 160.0 mm^2^, flip angle 60°, matrix: 384 × 384, slice thickness 3.0 mm.

**Fig. 1:**
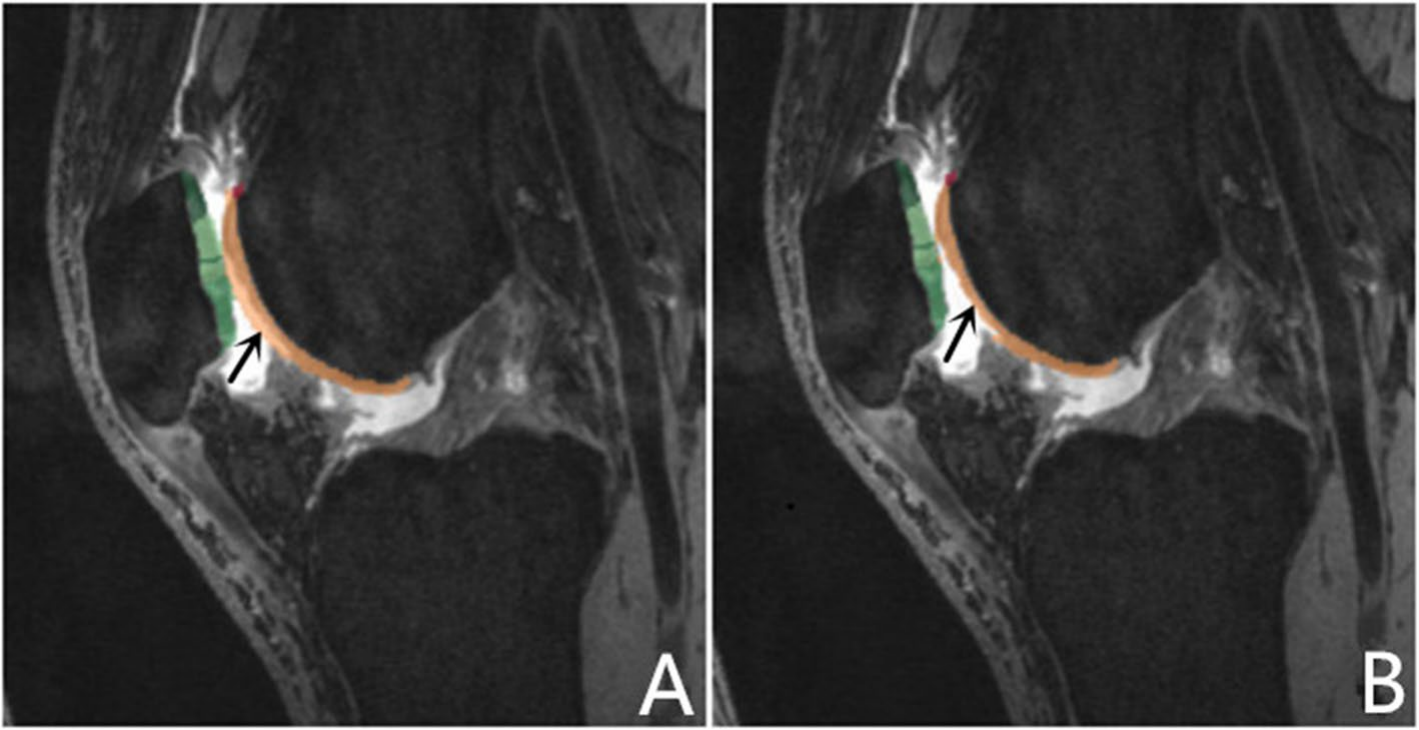
Cartilage segmentation: automated (**A**) vs automated plus manual correction automated (**B**): due to joint effusion, automatic segmentation identifies joint effusion as articular cartilage in the trochlea central and lateral of femur (black arrow)

A senior-level radiologist, who was blinded to the volunteers’ clinical information, evaluated the extent of cartilage degeneration and hydrarthrosis. Cartilage score criteria was obtained using the Whole-Organ Magnetic Resonance Imaging Score (WORMS) and ranged from 0 to 2. Knee cartilage was automatically segmented into 21 subregions [14] using post-processing prototype software (MR Chondral Health, version 2.1, Siemens Healthcare, Erlangen, Germany). This software automatically divides the knee cartilage into three main parts—femoral, patellar, and tibial cartilage—consisting of 21 cartilage subregions. The T2* maps were automatically registered to 3D DESS images by prototype software. The cartilage volume and mean T2* relaxation time for each subregion were also derived automatically by the software. The corrected slice was the slice that needs to be manually adjusted after automatic segmentation (Fig. 1). The T2* relaxation time of cartilage in the knee was measured by the same doctor twice a week apart to test consistency among observers. The automatic segmentations were manually corrected to increase overall segmentation accuracy. The Dice coefficient was used to quantify the amount of change performed on the automatic segmentation [5, 11]. The Dice correlation between automatic segmentation A (fA(x)) and manual correction based on the automatic segmentation B (fB(x)) was defined as in Equation: Dice (fA(x),fB(x)) = 2·fA(x)·fB(x)/(fA(x) + fB(x)). Levels of hydrarthrosis and cartilage scores (by WORMS) were determined to analyze their influence on the segmentation accuracy of each cartilage subregion.

Statistical analysis was performed with SPSS v.17.0 (IBM, Chicago, IL) and was expressed as mean ± standard deviation (Tables 1, 2). *P* values below 0.05 were considered to be statistically significant. Due to the small sample size, the paired rank sum test and independent sample *t* test were used to compare the regional differences of T2* relaxation parameter between the automatic segmentation and manual correction based on automatic segmentation in different groups by articular effusion and cartilage degeneration (Table 2).

**Table 1:** The cartilage mean volume ± standard deviation (SD), regional differences and Dice similarity coefficients between the automatic segmentation and manual correction based on automatic segmentation in different groups by articular effusion and cartilage degeneration

Sub-region		ALL (*n* = 32)			No hydrarthrosis(*n* = 14)			Hydrarthrosis (*n* = 18)			WORMS 0 (*n* = 21)			WORMS 1–2 (*n* = 11)		
		mean ± SD (mL)	P	Dice	mean ± SD (mL)	P	Dice	mean ± SD (mL)	P	Dice	mean ± SD (mL) P Dice			mean ± SD (mL) P Dice		
FMP	A	0.64 ± 0.16		1.0000	0.61 ± 0.17		1.0000	0.67 ± 0.16		1.0000	0.59 ± 0.13		1.0000	0.73 ± 0.18		1.0000
	M	0.64 ± 0.16			0.61 ± 0.17			0.66 ± 0.16			0.59 ± 0.13			0.73 ± 0.18		
FMC	A	1.08 ± 0.21	0.001	0.9978	1.02 ± 0.22	0.026	0.9975	1.12 ± 0.21	0.003	0.9979	0.99 ± 0.14	0.001	0.9971	1.25 ± 0.24	0.083	0.9992
	M	1.07 ± 0.22			1.01 ± 0.22			1.12 ± 0.21			0.98 ± 0.14			1.25 ± 0.24		
FMA	A	0.98 ± 0.18	0.001	0.9958	0.92 ± 0.17	0.004	0.9960	1.03 ± 0.18	0.001	0.9957	0.94 ± 0.14	0.001	0.9955	1.07 ± 0.23	0.018	0.9966
	M	0.97 ± 0.18			0.91 ± 0.18			1.01 ± 0.17			0.92 ± 0.14			1.06 ± 0.22		
FTM	A	0.90 ± 0.18	0.046	0.9994	0.86 ± 0.20	0.317	0.9993	0.93 ± 0.15	0.083	0.9995	0.86 ± 0.15	0.083	0.9992	0.98 ± 0.21	0.317	0.9999
	M	0.90 ± 0.18			0.86 ± 0.20			0.93 ± 0.16			0.85 ± 0.15			0.98 ± 0.20		
FTC	A	1.76 ± 0.40	0.001	0.9935	1.65 ± 0.43	0.007	0.9965	1.84 ± 0.37	0.003	0.9914	1.72 ± 0.43	0.001	0.9961	1.82 ± 0.36	0.027	0.9878
	M	1.73 ± 0.40			1.63 ± 0.43			1.81 ± 0.36			1.70 ± 0.43			1.78 ± 0.33		
FTL	A	1.76 ± 0.32	0.001	0.9945	1.63 ± 0.27	0.004	0.9930	1.86 ± 0.34	0.002	0.9956	1.67 ± 0.31	0.001	0.9943	1.92 ± 0.31	0.017	0.9951
	M	1.73 ± 0.33			1.60 ± 0.29			1.84 ± 0.32			1.65 ± 0.32			1.89 ± 0.28		
FLP	A	0.63 ± 0.13		1.0000	0.61 ± 0.15		1.0000	0.65 ± 0.10		1.0000	0.63 ± 0.14		1.0000	0.63 ± 0.10		1.0000
	M	0.63 ± 0.13			0.61 ± 0.15			0.65 ± 0.10			0.63 ± 0.14			0.63 ± 0.10		
FLC	A	1.06 ± 0.22	0.317	1.0000	1.00 ± 0.26		1.0000	1.11 ± 0.18	0.317	1.0000	0.97 ± 0.18	0.317	1.0000	1.23 ± 0.18		1.0000
	M	1.06 ± 0.22			1.00 ± 0.26			1.11 ± 0.18			0.98 ± 0.18			1.23 ± 0.18		
FLA	A	1.05 ± 0.25	0.102	0.9994	0.96 ± 0.19	0.157	0.9991	1.12 ± 0.27	0.317	0.9995	0.96 ± 0.20	0.180	0.9992	1.21 ± 0.26	0.317	0.9998
	M	1.05 ± 0.25			0.96 ± 0.19			1.12 ± 0.27			0.96 ± 0.20			1.21 ± 0.26		
PLI	A	0.26 ± 0.08	0.134	0.9860	0.23 ± 0.06	0.083	0.9902	0.28 ± 0.08	0.235	0.9831	0.25 ± 0.08	0.679	0.9830	0.27 ± 0.08	0.042	0.9927
	M	0.25 ± 0.07			0.23 ± 0.07			0.27 ± 0.07			0.25 ± 0.07			0.25 ± 0.057		
PLC	A	0.65 ± 0.14	0.001	0.9836	0.64 ± 0.14	0.008	0.9871	0.66 ± 0.15	0.010	0.9812	0.66 ± 0.16	0.001	0.9812	0.63 ± 0.09	0.063	0.9889
	M	0.64 ± 0.14			0.63 ± 0.14			0.66 ± 0.15			0.66 ± 0.17			0.63 ± 0.09		
PLS	A	0.19 ± 0.06		0.9932	0.17 ± 0.05		0.9923	0.21 ± 0.07		0.9938	0.19 ± 0.07		0.9917	0.17 ± 0.04		0.9964
	M	0.19 ± 0.06			0.17 ± 0.05			0.21 ± 0.07			0.19 ± 0.07			0.19 ± 0.04		
PMI	A	0.43 ± 0.12	0.003	0.9885	0.42 ± 0.07	0.005	0.9953	0.44 ± 0.15	0.095	0.9839	0.41 ± 0.07	0.048	0.9957	0.48 ± 0.18	0.01	0.9727
	M	0.42 ± 0.11			0.40 ± 0.06			0.44 ± 0.14			0.40 ± 0.06			0.46 ± 0.18		
PMC	A	0.94 ± 0.16	0.001	0.9923	0.94 ± 0.19	0.002	0.9925	0.94 ± 0.14	0.002	0.9921	0.95 ± 0.14	0.001	0.9913	0.91 ± 0.20	0.01	0.9945
	M	0.91 ± 0.17			0.92 ± 0.19			0.91 ± 0.14			0.92 ± 0.14			0.90 ± 0.20		
PMS	A	0.40 ± 0.11	0.001	0.9995	0.38 ± 0.08	0.066	0.9999	0.41 ± 0.13	0.006	0.9993	0.41 ± 0.12	0.004	0.9993	0.37 ± 0.09	0.083	1.0000
	M	0.39 ± 0.11			0.38 ± 0.08			0.40 ± 0.13			0.40 ± 0.11			0.36 ± 0.09		
TLP	A	0.84 ± 0.23	0.317	1.0000	0.79 ± 0.05	0.317	1.0000	0.87 ± 0.26		1.0000	0.79 ± 0.24	0.317	1.0000	0.92 ± 0.20		1.0000
	M	0.84 ± 0.23			0.80 ± 0.05			0.87 ± 0.26			0.80 ± 0.23			0.92 ± 0.20		
TLC	A	1.05 ± 0.23	0.046	0.9997	1.02 ± 0.22	0.157	0.9994	1.07 ± 0.24	0.157	0.9999	1.00 ± 0.21	0.046	0.9996	1.15 ± 0.23		0.9998
	M	1.05 ± 0.23			1.02 ± 0.22			1.07 ± 0.24			0.99 ± 0.22			1.15 ± 0.23		
TLA	A	0.52 ± 0.15	0.317	0.9998	0.48 ± 0.15		1.0000	0.55 ± 0.15	0.317	0.9997	0.49 ± 0.16		1.0000	0.58 ± 0.12	0.317	0.9994
	M	0.52 ± 0.15			0.48 ± 0.15			0.55 ± 0.15			0.4895 ± 0.1614			0.58 ± 0.12		
TMP	A	0.52 ± 0.09	0.046	0.9993	0.52 ± 0.11	0.317	0.9994	0.52 ± 0.08	0.083	0.9991	0.5148 ± 0.0889	0.046	0.9990	0.53 ± 0.10		0.9997
	M	0.52 ± 0.09			0.52 ± 0.11			0.52 ± 0.08			0.51 ± 0.09			0.53 ± 0.10		
TMC	A	0.85 ± 0.17	0.001	0.9972	0.82 ± 0.18	0.010	0.9974	0.87 ± 0.16	0.004	0.9971	0.80 ± 0.16	0.001	0.9970	0.93 ± 0.16	0.102	0.9978
	M	0.84 ± 0.17			0.81 ± 0.18			0.86 ± 0.16			0.79 ± 0.16			0.92 ± 0.16		
TMA	A	0.54 ± 0.12	0.001	0.9975	0.49 ± 0.09	0.002	0.9965	0.58 ± 0.13	0.002	0.9982	0.49 ± 0.10	0.001	0.9974	0.63 ± 0.12	0.01	0.9978
	M	0.52 ± 0.13			0.46 ± 0.09			0.56 ± 0.14			0.47 ± 0.10			0.62 ± 0.12		
V	A	17.03 ± 3.11	0.001	0.9962	16.15 ± 3.20	0.001	0.9966	17.72 ± 2.95	0.001	0.9959	16.29 ± 2.90	0.001	0.9960	18.46 ± 3.14	0.003	0.9965
	M	16.87 ± 3.08			16.00 ± 3.20			17.54 ± 2.88			16.13 ± 2.91			18.29 ± 3.02		

*Dice* Dice similarity coefficient, A Automatic cartilage segmentation group, *M* Manual correction based on automatic segmentation group, *V* Volume of cartilage, *FMP* Femoral medial posterior, *FMC* Femoral medial central, *FMA* Femoral medial anterior, *FTM* Femoral trochlea medial, *FTC* Femoral trochlea central, *FTL* Femoral trochlea lateral, *FLP* Femoral lateral posterior, *FLC* Femoral lateral central, *FLA* Femoral lateral anterior, *PLI* Patellar lateral inferior, *PLC* Patellar lateral central, *PLS* Patellar lateral superior, *PMI* Patellar medial inferior, *PMC* Patellar medial central, *PMS* Patellar medial superior, *TLP* Tibial lateral posterior, *TLC* Tibial lateral central, *TLA* Tibial lateral anterior, *TMP* Tibial medial posterior, *TMC* Tibial medial central, *TMA* Tibial medial anterior

**Table 2:** The cartilage mean T2* value ± standard deviation (SD) and regional differences between the automatic segmentation and manual correction based on automatic segmentation in different groups by articular effusion and cartilage degeneration

Subregion		ALL(*n* = 32)		No hydrarthrosis(*n* = 14)		Hydrarthrosis(*n* = 18)		WORMS 0 (*n* = 21)		WORMS 1–2 (*n* = 11)	
		Mean ± SD (ms)	*P*	Mean ± SD (ms)	*P*	Mean ± SD (ms)	*P*	Mean ± SD (ms)	*P*	Mean ± SD (ms)	*P*
FMP	A	26.58 ± 2.94		27.73 ± 2.77		25.68 ± 2.83		26.37 ± 3.15		26.97 ± 2.59	
	M	26.58 ± 2.95		27.73 ± 2.77		25.68 ± 2.83		26.37 ± 3.15		26.97 ± 2.59	
FMC	A	27.06 ± 3.60	0.001	26.20 ± 2.55	0.008	27.72 ± 4.20	0.001	26.85 ± 3.51	0.001	27.45 ± 3.93	0.012
	M	26.58 ± 3.66		25.62 ± 2.78		27.32 ± 4.14		26.19 ± 3.54		27.31 ± 3.93	
FMA	A	26.33 ± 4.44	0.001	24.77 ± 4.46	0.001	27.55 ± 4.15	0.001	27.04 ± 3.83	0.001	24.98 ± 5.37	0.005
	M	25.39 ± 3.97		24.10 ± 4.33		26.39 ± 3.46		25.92 ± 3.23		24.37 ± 5.13	
FTM	A	27.89 ± 5.30	0.003	26.00 ± 2.45	0.028	29.35 ± 6.43	0.046	25.89 ± 2.38	0.008	31.71 ± 7.15	0.285
	M	27.81 ± 5.31		25.95 ± 2.47		29.26 ± 6.45		25.78 ± 2.32		31.68 ± 7.17	
FTC	A	35.83 ± 14.93	0.001	30.37 ± 4.41	0.002	40.07 ± 18.65	0.001	31.90 ± 4.04	0.001	43.33 ± 23.74	0.008
	M	31.98 ± 6.54		29.09 ± 3.33		34.23 ± 7.56		30.20 ± 2.87		35.38 ± 9.82	
FTL	A	32.57 ± 7.13	0.001	32.85 ± 6.79	0.002	32.35 ± 7.57	0.001	31.50 ± 6.20	0.001	34.61 ± 8.59	0.008
	M	29.73 ± 3.38		29.24 ± 1.71		30.10 ± 4.28		28.70 ± 2.40		31.69 ± 4.17	
FLP	A	24.79 ± 2.88		24.72 ± 3.19		24.84 ± 2.70		24.86 ± 2.98		24.65 ± 2.79	
	M	24.79 ± 2.88		24.73 ± 3.19		24.84 ± 2.70		24.86 ± 2.98		24.65 ± 2.79	
FLC	A	28.65 ± 4.02	0.109	27.77 ± 4.44	0.109	29.34 ± 3.63		27.70 ± 3.29	0.109	30.47 ± 4.78	
	M	28.64 ± 4.01		27.74 ± 4.4		29.34 ± 3.63		27.69 ± 3.27		30.47 ± 4.78	
FLA	A	26.67 ± 5.56	0.018	26.15 ± 6.28	0.109	27.07 ± 5.09	0.068	26.55 ± 5.53	0.043	26.89 ± 5.88	0.180
	M	26.57 ± 5.57		26.09 ± 6.30		26.95 ± 5.09		26.44 ± 5.54		26.84 ± 5.91	
PLI	A	29.56 ± 19.81	0.001	22.92 ± 5.69	0.049	34.72 ± 25.02	0.008	23.97 ± 4.39	0.008	40.23 ± 31.41	0.049
	M	23.86 ± 5.22		22.40 ± 5.64		25.00 ± 4.71		23.42 ± 4.48		24.70 ± 6.43	
PLC	A	25.52 ± 5.47	0.001	24.47 ± 5.21	0.002	26.34 ± 5.67	0.001	25.30 ± 4.35	0.001	27.85 ± 6.77	0.008
	M	23.97 ± 4.31		22.87 ± 3.93		24.82 ± 4.51		22.97 ± 3.51		25.88 ± 5.18	
PLS	A	23.11 ± 4.13	0.317	22.30 ± 3.22		23.74 ± 4.71	0.317	22.61 ± 4.24	0.317	24.05 ± 3.91	
	M	23.10 ± 4.12		22.30 ± 3.22		23.72 ± 4.70		22.60 ± 4.23		24.05 ± 3.91	
PMI	A	30.15 ± 6.90	0.001	28.06 ± 6.26	0.001	31.77 ± 7.10	0.001	29.86 ± 6.34	0.001	30.68 ± 8.17	0.005
	M	27.21 ± 4.66		25.74 ± 4.21		28.35 ± 4.79		26.95 ± 4.78		27.70 ± 4.61	
PMC	A	32.67 ± 7.02	0.001	30.59 ± 6.19	0.001	34.287.36	0.001	32.30 ± 7.26	0.001	33.37 ± 6.81	0.008
	M	30.13 ± 4.94		29.68 ± 6.24		30.48 ± 3.81		29.11 ± 3.80		32.07 ± 6.38	
PMS	A	26.68 ± 6.17	0.001	27.57 ± 5.46	0.028	25.98 ± 6.74	0.001	26.35 ± 6.16	0.002	27.30 ± 6.44	0.018
	M	25.54 ± 4.80		26.61 ± 5.66		24.71 ± 3.99		24.87 ± 3.84		26.83 ± 6.27	
TLP	A	23.10 ± 2.50		22.33 ± 2.02		23.69 ± 2.73		22.37 ± 2.29		24.49 ± 2.39	
	M	23.10 ± 2.50		22.33 ± 2.02		23.69 ± 2.73		22.37 ± 2.29		24.49 ± 2.39	
TLC	A	20.84 ± 3.05	0.046	21.96 ± 2.86	0.285	19.98 ± 2.98	0.109	20.65 ± 3.24	0.043	21.20 ± 2.76	0.317
	M	20.77 ± 2.96		21.90 ± 2.76		19.88 ± 2.87		20.54 ± 3.09		21.20 ± 2.76	
TLA	A	24.75 ± 7.27	0.180	24.09 ± 5.98		25.26 ± 8.28	0.18	23.04 ± 4.88	0.317	28.01 ± 9.93	0.317
	M	24.32 ± 6.14		24.09 ± 5.98		24.49 ± 6.42		22.84 ± 4.68		27.14 ± 7.72	
TMP	A	18.77 ± 2.34	0.001	17.88 ± 2.03	0.013	19.47 ± 2.39	0.009	18.13 ± 1.88	0.001	20.00 ± 2.72	0.18
	M	18.63 ± 2.34		17.80 ± 1.97		19.28 ± 2.44		17.94 ± 1.85		19.94 ± 2.68	
TMC	A	19.51 ± 3.74	0.001	20.28 ± 4.01	0.001	18.91 ± 3.51	0.001	18.65 ± 3.06	0.001	21.16 ± 4.49	0.011
	M	18.63 ± 2.34		19.20 ± 1.90		18.58 ± 3.34		18.25 ± 2.76		19.99 ± 2.55	
TMA	A	23.93 ± 6.56	0.001	25.10 ± 8.07	0.002	23.01 ± 5.17	0.002	23.50 ± 7.36	0.001	24.73 ± 4.91	0.012
	M	21.86 ± 5.32		22.28 ± 6.80		21.53 ± 3.99		21.35 ± 5.50		22.82 ± 5.05	
T	A	21.82 ± 5.13	0.001	21.94 ± 5.16	0.001	21.72 ± 5.13	0.001	21.06 ± 4.63	0.001	23.26 ± 5.73	0.001
	M	21.25 ± 4.43		21.27 ± 4.51		21.24 ± 4.39		20.55 ± 4.01		22.60 ± 4.90	

*A* Automatic cartilage segmentation group, *M* Manual correction based on automatic segmentation group, *T* Total cartilage, *FMP* Femoral medial posterior, *FMC* Femoral medial central, *FMA* Femoral medial anterior, *FTM* Femoral trochlea medial, *FTC* Femoral trochlea central, *FTL* Femoral trochlea lateral, *FLP* Femoral lateral posterior, *FLC* Femoral lateral central, *FLA* Femoral lateral anterior, *PLI* Patellar lateral inferior, *PLC* Patellar lateral central, *PLS* Patellar lateral superior, *PMI* Patellar medial inferior, *PMC* Patellar medial central, *PMS* Patellar medial superior, *TLP* Tibial lateral posterior, *TLC* Tibial lateral central, *TLA* Tibial lateral anterior, *TMP* Tibial medial posterior, *TMC* Tibial medial central, *TMA* Tibial medial anterior

## Results

The intra-observer correlation coefficient was 0.99 for T2* measurement. The manual correction based on the automatic segmentation was commonly done in FMC, FMA, FTM, FTC, FTL, PLC, PMI, PMC, PMS, TLC, TLP, TMP, TMC, and TMA. Cartilage volume in the manual corrected group was less than in the automatic cartilage segmentation group (*P* < 0.05). Table 1 lists cartilage volume, *P* values, and Dice coefficient correlations for each subregion. In FMC, FMA, FTM, FTC, FTL, FLA, PLI, PLC, PMI, PMC, PMS, TLC, TMP, TMC and TMA subregions, the T2* relaxation value of manual corrected cartilage segmentation group was less than that of automatic cartilage segmentation group (*P* < 0.05). The Dice correlations between automatic segmentation and manual correction in different groups by articular effusion and cartilage degeneration are shown in Table 1. The mean Dice coefficient in FMP, FLP, FLC, TLP and TLA was close to 1, indicating an already high accuracy of the automatic segmentation prior to manual refinement. Cartilage T2* values and regional dissimilarities of all the subregions in the two groups are shown in Table 2.

**Fig. 2:**
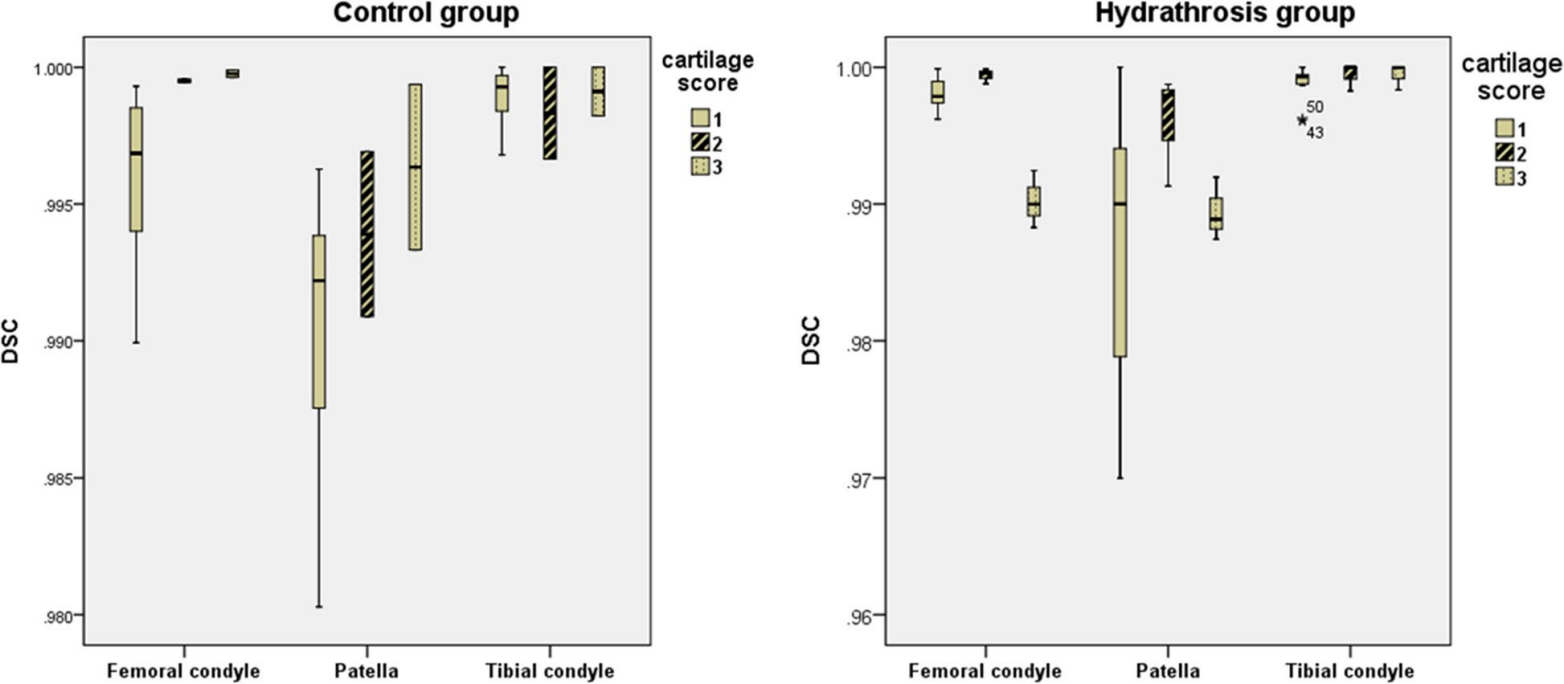
Cartilage score influence on Dice coefficient of the automatic cartilage segmentation software in the control and hydrarthrosis groups. Hydrarthrosis significantly decreased the Dice coefficient of moderate degenerated patellar cartilage and distal femoral cartilage but increased the Dice coefficient of mild degenerated proximal tibia cartilage. (WORMS 0 = normal; 1 = mild; 2 = moderate)

With the increase in joint effusion, the Dice coefficient in the patella increased somewhat, but the difference was not statistically significant. The femoral condyle and patella had lower Dice coefficient (0.9969 and 0.9922, respectively) than the other regions of the knee cartilage when the cartilage score was 0 in the control group. The femoral condyle and patella had the lowest Dice coefficient (0.9900 and 0.9889, respectively) when the cartilage score was 2 in the hydrarthrosis group (Fig. 2).

The average time for the automatic segmentation software to complete cartilage segmentation of a knee was around 6 min (for a processor model), while the average time for manual correction of a knee was around 27 min.

## Discussion

These results suggest that use of the automated segmentation software results in a Dice coefficient of each subregion of higher than 0.9. The location of subregions, extent of hydrarthrosis, and level of cartilage degeneration are the most important factors affecting the accuracy of automatic segmentation. Automatic segmentation software can mistake some of the fluid accumulation at the edges of subregions, resulting in an overestimate of cartilage volume. These areas deserve greater attention during manual correction to increase segmentation accuracy in these parts. In all 21 subregions, the subregions with the most corrected slices were located in the medial anterior, central trochlea and lateral trochlea of the femoral condyle, medial inferior, and medial central of the patellar, and the medial anterior of the tibia condyle. These subregions were likely influenced by hydrarthrosis. Under the influence of hydrarthrosis, the Dice coefficient for automatic segmentation of the femoral condyle and patellar cartilage decreased when the cartilage score was 2.

The evaluation of T2* has been shown to be capable of characterizing different degrees of cartilage degeneration [15]. It had been proposed as a robust biomarker of articular cartilage degeneration in several joints [16, 17]. The advantages compared to T2 mapping include shorter scan times and higher SNR [5]. In this study, the presence of hydrarthrosis and a higher cartilage degeneration score decreased T2*. According to the literature, T2* may be susceptible to the spatial macromolecule architecture and its influence on water molecule mobility [5, 18]. In this study, failure of the automatic segmentation software to distinguish the contour of the cartilage occurred mainly in articular cartilage near the fluid accumulation. A segmentation algorithm with increased robustness against synovial fluid is currently being integrated, but was not available for testing at the time of this study. The boundary between articular effusion and articular cartilage was not clearly visible. The T2* relaxation times of cartilage subregions extracted with manually corrected segmentation was decreased compared to those extracted with automatic segmentation. Articular effusion was recognized as articular cartilage by the automatic segmentation, resulting in the increase of the T2* relaxation times in the uncorrected subregions.

There some limitations in this study. Although only normal volunteers were included, some undiagnosed cartilage degeneration was present. The accuracy of the segmentation of degenerated cartilage needs further evaluation. In addition, a larger sample size is required to increase reliability of results. A further limitation of this study was lack of inter-observer variability assessment for manually corrected faulty segmentations.

## Conclusions

In general, automatic cartilage segmentation software had a high Dice coefficient and it can accurately evaluate the volume of cartilage. It provides quantitative information about cartilage morphology within an acceptable time range usually less than 10 min even on a laptop.

Manual correction can be used to improve the accuracy of the segmentation. The location of cartilage subregions and extent of hydrarthrosis and cartilage degeneration may influence segmentation accuracy. To derive exact results of T2* relaxation times of cartilage, manual correction of automatic segmentation is necessary, but even then using the software saves considerable time.

## Data Availability

Part of the statistical results of this study have not been published, so the data set cannot be deposited at present. If you have special needs, please contact the corresponding author.

## Abbreviations

3D: Three-dimensional
DESS: Double-Echo in Steady-State
WORMS: Whole-Organ Magnetic Resonance Imaging Score
MRI: Magnetic resonance imaging
CNN: Convolutional neural network
FMP: Femoral medial posterior
FMC: Femoral medial central
FMA: Femoral medial anterior
FTM: Femoral trochlea medial
FTC: Femoral trochlea central
FTL: Femoral trochlea lateral
FLP: Femoral lateral posterior
FLC: Femoral lateral central
FLA: Femoral lateral anterior
PLI: Patellar lateral inferior
PLC: Patellar lateral central
PLS: Patellar lateral superior
PMI: Patellar medial inferior
PMC: Patellar medial central
PMS: Patellar medial superior
TLP: Tibial lateral posterior
TLC: Tibial lateral central
TLA: Tibial lateral anterior
TMP: Tibial medial posterior
TMC: Tibial medial central
TMA: Tibial medial anterior
